# *Cryptococcus gattii* Dispersal Mechanisms, British Columbia, Canada

**DOI:** 10.3201/eid1301.060823

**Published:** 2007-01

**Authors:** Sarah E. Kidd, Paxton J. Bach, Adrian O. Hingston, Sunny Mak, Yat Chow, Laura MacDougall, James W. Kronstad, Karen H. Bartlett

**Affiliations:** * University of British Columbia, Vancouver, British Columbia, Canada; † British Columbia Centre for Disease Control, Vancouver, British Columbia, Canada

**Keywords:** Cryptococcus gattii, dispersal, colonization, British Columbia, Pacific Northwest, research

## Abstract

Recent *Cryptococcus gattii* infections in humans and animals without travel history to Vancouver Island, as well as environmental isolations of the organism in other areas of the Pacific Northwest, led to an investigation of potential dispersal mechanisms. Longitudinal analysis of *C. gattii* presence in trees and soil showed patterns of permanent, intermittent, and transient colonization, reflecting *C. gattii* population dynamics once the pathogen is introduced to a new site. Systematic sampling showed *C. gattii* was associated with high-traffic locations. In addition, *C. gattii* was isolated from the wheel wells of vehicles on Vancouver Island and the mainland and on footwear, consistent with anthropogenic dispersal of the organism. Increased levels of airborne *C. gattii* were detected during forestry and municipal activities such as wood chipping, the byproducts of which are frequently used in park landscaping. *C. gattii* dispersal by these mechanisms may be a useful model for other emerging pathogens.

## Abstract 2

*C. gattii* may be spread through soil disturbances, wind, water, distribution of tree and soil byproducts, and human movement.

The basidiomycete fungal pathogen *Cryptococcus gattii* can infect the pulmonary and central nervous systems of humans and animals and was until recently regarded as a predominantly tropical organism (*1*,*2*). *C. gattii* began to emerge as a primary pathogen on Vancouver Island, British Columbia (BC), in 1999 (*3*). Most BC *C. gattii* cases were among humans or animals that had contact with the Coastal Douglas Fir and Coastal Western Hemlock xeric maritime biogeoclimatic zones of Vancouver Island (*3*–*5*). However, a number of infections in humans and animals with no travel to *C. gattii–*endemic areas were recently confirmed on the BC mainland and in Washington and Oregon in the United States (*6*), indicating dispersal within the Pacific Northwest. Strains of the *C. gattii* VGIIa genotype were isolated on 3 Gulf Islands that are clustered with the San Juan Islands between Vancouver Island and the mainland, as well from air samples on the BC lower mainland, and from soil and a fencepost in northern Washington (*6*). These findings indicated that, in contrast to a previous report (*7*), the Strait of Georgia/Juan de Fuca does not form a geographic barrier to *C. gattii* dispersal and that mechanisms for the dispersal of *C. gattii* exist in the Pacific Northwest. A recent gene genealogy study found evidence for global dispersal of *C. gattii* (*8*).

A large-scale study of the environmental distribution of *C. gattii* in the Pacific Northwest showed that focal areas were characterized by comparatively high concentrations (Kidd et al., unpub. data). With the exception of 1 Gulf Island location with extremely high *C. gattii* concentrations in soil, these “hotspot” areas were all on Vancouver Island, which suggests that this is the primary area of *C. gattii* colonization. Approximately 10% of trees were positive for *C. gattii,* including >10 species. The highest airborne *C. gattii* concentrations were detected during the Northern Hemisphere summer but with propagules sufficiently small to cause infection present throughout the year. *C. gattii* was detected in fresh water and seawater in several locations.

While the association between *C. gattii* and exported tree species, particularly eucalypts, has been speculatively linked to its dispersal (*6*,*9*–*11*), no evidence for this has been found in BC. We investigated potential mechanisms for *C. gattii* dispersal and transmission within the Pacific Northwest region, given recent indications of an expanding distribution. We addressed the potential for *C. gattii* mobility in the environment through distribution of tree byproducts, aerosolization, water flow, and anthropogenic factors. An improved understanding of the mechanisms of dispersal and the risk of exposure to *C. gattii* could facilitate a model to effectively manage the emergence of cryptococcal and other infectious diseases in previously non–disease-endemic areas.

## Materials and Methods

### Environmental Sampling Strategies

Trees, soil, debris, wood chips, water, and air samples were collected according to previously described techniques, with limits of detection as previously described (Kidd et al., unpub. data). Sampling was conducted in the environments surrounding the residences of those with reported infections, including homes, habitats, and nearby parks and wooded areas.

A 35-km traffic corridor connecting 2 highly visited provincial parks was sampled at ≈500-m intervals (publicly accessible areas only). This sampling was performed on 6 nonconsecutive days in October 2004 and included 92 sites, 64 located at the roadside (designated “road sites”) and 28 located ≈100 m from the road (designated “forest sites”). For this series of samples, 169 trees of 8 different species were swabbed, and soil samples were collected from the rhizospheres of 77 of these trees. Global positioning system coordinates and tags were used to identify trees. Sites were designated positive by the presence of at least 1 tree or soil sample positive for *C. gattii.* Positive sites were resampled in June and December of 2005, including the original positive tree(s) and adjacent trees.

Many sites were sampled multiple times within 2–3 years to investigate the longitudinal colonization patterns of *C. gattii* in the environment. Except in these analyses, all data presented consider only the first swab, soil, and air samples collected at each sampling point to minimize statistical artifacts.

To investigate the effect of forestry activities on *C. gattii* aerosolization, air samples were collected during 2 independent tree removal efforts in a *C. gattii*–endemic area of Vancouver Island. A red alder (*Alnus rubra*) and a Douglas fir (*Pseudotsuga menziesii* var. *menziesii*) tree were removed by arborists on the same day in August 2002. The trees were tested for *C. gattii* colonization by swab and air sampling immediately before felling; in addition, air samples were collected at different tree heights during felling.

In collaboration with a municipal garden waste removal service, swabs of garden waste and nearby trees were collected from properties on 17 residential streets in October 2003. Air samples were collected around the garden waste and at the outlet of the wood chipper. Samples of wood chips were also collected from the wood chipper.

The wheel wells of vehicles used for Vancouver Island sampling were routinely swabbed; 63 swabs have been taken since July 2003. In addition, wheel wells of privately owned mainland- and Vancouver Island–based vehicles were swabbed to evaluate *C. gattii* carriage on vehicles not involved in the sampling effort. Two hundred vehicles in a mainland university campus parking lot and 200 vehicles in a Vancouver Island university campus parking lot were randomly selected for testing.

Eighty swabs of footwear worn by personnel participating in *C. gattii* sampling were taken at various Vancouver Island sampling sites during July 2003–June 2004. In addition, 9 swabs were taken from surfaces that came in contact with *C. gattii*–positive footwear (i.e., carpet, steel flooring, and a plastic bag). To investigate the potential for survival of *C. gattii* on footwear, shoes worn in a *C. gattii*–endemic area of Vancouver Island were stored for 333 days, with swabs taken periodically. The shoes were worn in nonendemic areas for short periods (≈4 h) on days 144, 153, and 154.

### Identification and Genotyping of *C. gattii* Isolates

Cryptococci were initially identified by using Staib agar (*12*). Isolates were subcultured on malt extract agar and confirmed as *C. gattii* by using canavanine-glycine-bromothymol blue media (*13*), serotyping, or both (Iatron Laboratories, Tokyo, Japan). Sample positivity was scored both binarily and by the detected *C. gattii* concentration. Swab concentration was estimated by the presence of colonies on progressive streak lines, on a scale of 0 to 4+. The molecular types of selected isolates were identified by using previously described restriction fragment length polymorphism (RFLP) methods (*6*,*14*).

### Data Analyses

Environmental sampling data was compiled by using Microsoft (Redmond, WA, USA) Access 2002, and statistical analyses were performed with SPSS 14 (SPSS Inc., Chicago, IL, USA). Geographic data were assembled, and sampling points were mapped on 1:50,000 scale National Topographic System of Canada (NTS) grids (Kidd et al., unpub. data).

## Results and Discussion

### Patterns of *C. gattii* Colonization

**Figure 1:**
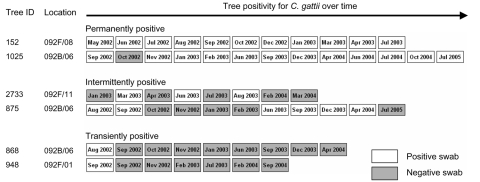
Example of longitudinal swab sampling profiles from trees designated permanently, intermittently, or transiently colonized with *Cryptococcus gattii*. Samples were collected during a 3-year period.

We initially investigated longitudinal patterns of *C. gattii* tree and soil colonization. Figure 1 illustrates these colonization patterns. Consistently positive swab and soil results were observed for some trees and their rhizospheres over 2–3 years, and these were designated “permanently colonized.” For other trees, an initial positive swab result was followed by a series of negative samples, which indicated a transient presence of *C. gattii* in these locations. Intermittently positive swab and soil results were also observed, in which the series of samples effectively oscillated between positive and negative for *C. gattii* over time with no discernable seasonal pattern. This intermittent positivity was probably due to fluctuations in the cryptococcal population over time, above and below limits of detection. This situation might arise following the introduction of *C. gattii* to a new location or substrate.

We hypothesize that permanent colonization is established once the cryptococcal population reaches a critical mass. This concept forms the basis of a model for *C. gattii* introduction to new areas of the Pacific Northwest, in which the fungus must adapt to new microclimates and compete with local microbiota. Areas of recent *C. gattii* dispersal may go through a period of intermittent positivity before either failing to become colonized (transience) or becoming permanently colonized. Determinants of colonization resulting in high *C. gattii* concentrations include low soil moisture and organic carbon content (Kidd et al., unpub. data).

### *C. gattii* Dispersal

#### Human-mediated Dispersal

**Figure 2:**
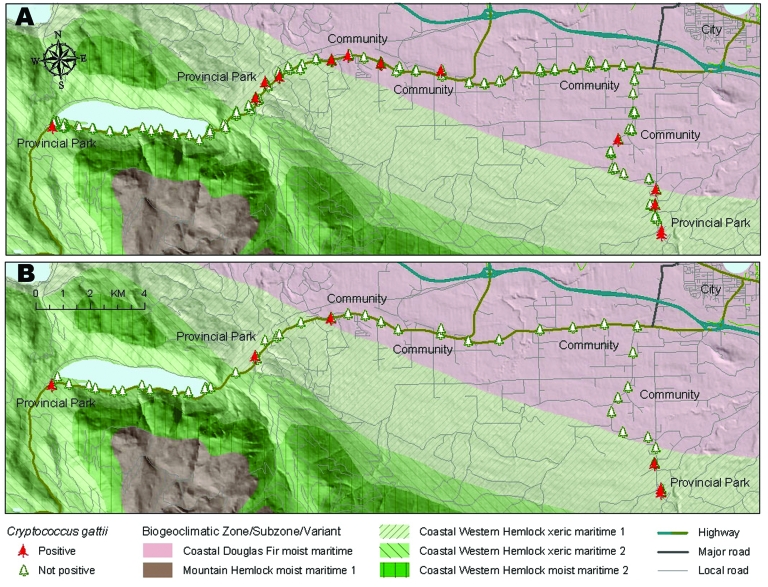
Distribution of positive and negative environmental samples for a systematic sampling along a 35-km traffic corridor traversing National Topographic System of Canada grids 092F/06 and 092F/07, highlighting transience of *Cryptococcus gattii* isolations. A) Sites of initial samples, collected in October 2004. B) Positive sites that were resampled in June 2005.

Using systematic sampling strategies, we acquired evidence of anthropogenic distribution of *C. gattii* in BC. We assessed *C. gattii* positivity at sites along a largely recreational traffic corridor that traverses both the Coastal Douglas Fir and Coastal Western Hemlock xeric maritime biogeoclimatic zones. Of the 169 trees sampled by swabbing on this route, 12 (7.1%) were positive for *C. gattii*, representing 10 sampling sites. Two of 77 soil samples (2.6%) from different sites were positive at low concentrations (25–50 CFU/g). No significant difference was observed between the *C. gattii* positivity of road sites and forest sites. Interestingly, the positive sampling sites were clustered at small towns, services, or attractions such as provincial parks (Figure 2). No sampling sites tested positive in areas of the sampling corridor where there was no apparent reason or safe location for a vehicle to pull off the road. These results support a model of *C. gattii* dispersal facilitated in part through human interaction with the environment.

In a resampling of trees and soil at these positive sites 8 months later, *C. gattii* was detected at only 6 of 12 previously positive sites (Figure 2). Specifically, only 2 of the 12 previously positive trees yielded positive swab results on the second sampling. Only 1 of the previously positive sampling sites remained positive at a third sampling in December 2005. This site, considered to be permanently positive, is located at the entrance to a highly visited provincial park (NTS grid 092F/08) where *C. gattii* is now regarded as endemic. RFLP genotyping indicated that all isolates from this sampling study belonged to the predominant genotype, VGIIa. Most trees and sampling sites in which *C. gattii* was initially detected appear to have been transiently or intermittently positive at that time, which is consistent with a model of recent dispersal, perhaps as a result of human activity.

Water sampling at a different provincial park (NTS grid 092F/07) also yielded evidence in support of anthropogenic *C. gattii* dispersal. The park contains a lake (≈550 km^2^) with a boat launch, hiking trails, and facilities for camping and picnics; 170,900 persons visited the park in 2005 (D. Forman, pers. comm.). Forty water samples were collected at 4 park sites during 11 separate sampling trips in a 32-month period, and at least 1 positive sample was obtained on each sampling trip. Table 1 provides a summary of the sampling sites at this lake and the *C. gattii* concentration detected at each. The boat launch site was associated with the highest rate of sample positivity and *C. gattii* concentration, followed by the picnic site and a historic artifact site, all of which are high-traffic visitor areas. Within the campground, water samples were collected from a creek feeding into the lake near the picnic site. These positive creek samples probably account for some of the *C. gattii* isolated from lake samples at the picnic site, although the observed *C. gattii* concentrations from creek samples were far lower than that of the lake samples. By contrast, 6 water samples taken in areas of the lake with limited public access were all negative for *C. gattii*. Many swab and soil samples collected from the public sites were positive for *C. gattii* (data not shown). While *C. gattii* in the water from these locations may be in part seeded by contaminated soil or tree debris, we suggest that human activities in and around the lake contributed to the dispersal of *C. gattii* to these recreational areas.

**Table 1:** *Cryptococcus gattii* positivity and concentration among water samples collected from different lake sites at a highly visited provincial park located within the NTS grid 092F/07*

Sampling site	Total samples	Positive samples (%)	*C. gattii* GM concentration (CFU/100 mL)†	GSD
Boat launch (lake)	11	10 (91)	11.6	10.6
Picnic site (lake)	9	6 (67)	4.9	2.8
Historic site (lake)	10	7 (70)	5.0	24.3
Campground (creek)	10	4 (40)	0.8	2.8
Other sites, limited public access (lake)	6	0	–	–

*NTS, National Topographic System of Canada; GM, geometric mean for positive samples; GSD, geometric standard deviation for positive samples. †Limit of detection of method: 0.2 CFU/100 mL (1 CFU/500 mL).

Sampling in other areas of BC detected *C. gattii* in several bodies of fresh water and seawater around Vancouver Island, and viability assays indicate the organism’s potential to survive for at least 1 year in fresh water and seawater (Kidd et al., unpub. data). These data provide some insight into the mechanism of transmission of cryptococcal infection reported for a considerable number of wild marine mammals in the Strait of Georgia (*4*,*15*).

The wheel wells of vehicles used for sampling were routinely swabbed to further investigate the role of humans in dispersal. *C. gattii* was detected in 22 (35%) of 63 samples, including samples taken several weeks after return of the vehicle to a non–*C. gattii*–endemic area, after the vehicle had been professionally washed. In addition, *C. gattii* was detected on 10 (5%) of 200 privately owned vehicles on Vancouver Island and 1 (0.5 %) of 200 on the BC mainland. The isolates obtained from the Vancouver Island–based vehicles represented molecular types VGIIa, VGIIb, and VGI (including coisolation of VGIIa and VGIIb from 1 vehicle), while the isolate from the mainland-based vehicle represented VGIIa. While we have not assessed the *C. gattii* positivity of cars that are actively traveling between Vancouver Island and the mainland, vehicles could certainly be involved in the mechanical dispersal of *C. gattii* propagules. Approximately 8 million private and commercial vehicles are transported between Vancouver Island and the BC mainland each year (*16*), and given the potential for extensive travel beyond these areas, dispersal of *C. gattii* in the Pacific Northwest likely can be attributed at least in part to the use of vehicles.

*C. gattii* was detected in 43 (54%) of 80 swab samples from the footwear of persons participating in sampling. In addition, 5 (56%) of 9 swabs of surfaces contacted by positive footwear were positive for *C. gattii*, which indicates that the fungus can be transferred to contacting surfaces and may be redistributed to some extent by this mechanism. Similarly, passive transport of *C. gattii* by wild and domestic animals could be involved in dispersal.

The viability of *C. gattii* carried on footwear was investigated over time. Swabs from shoes worn for environmental sampling were positive at day 0 and were consistently scored as 2+ when unworn (up to day 144). The active wearing of the shoes reduced viable *C. gattii* levels; *C. gattii* was detected following activity on day 144 but not after activity on days 153 and 154. However, viable levels rebounded slightly by day 183 (1+) and remained detectable on day 333 (1+). Genotyping of isolates from this footwear showed isolates belonging to the VGIIa, VGIIb, and VGI subtypes, reflecting the diversity observed at the sampling site where the shoes became contaminated on day 0 (data not shown). While different footwear materials and activity patterns are likely to influence viability and dispersal of *C. gattii*, these observations suggest that footwear could serve as mechanical vectors for *C. gattii*.

#### *C. gattii* Dispersal through Forestry Activity

The concentration of airborne *C. gattii* was investigated during the scheduled removal of 2 trees in an area of Vancouver Island where *C. gattii* had been found. Both trees tested positive for *C. gattii* by swab and by adjacent air sampling done immediately before they were felled. Table 2 shows the detected *C. gattii* concentrations in air samples taken during tree cutting, limb removal, and chipping activities. All air samples, collected at varying heights, tested positive during the felling of both trees. Airborne concentrations increased during felling of the red alder, but no substantial change was observed for the Douglas fir. However, for both trees, air samples taken during branch chipping indicated much greater (10- to 140-fold) airborne *C. gattii* concentrations than were observed during quiescence. Aerosolization of *C. gattii* through such activities is likely to increase the risk of exposure and the dispersal of cryptococci through wind.

**Table 2:** Airborne *Cryptococcus gattii* concentration before and during contracted tree-cutting activities

Tree-cutting activity	Sampling method	*C. gatti*i concentration in air (CFU/m^3^)	
		Red alder	Douglas fir
Quiescent	Swab	Positive	Positive
	Air; Andersen 6-stage*	381	2,073
Limb removal	Air; Andersen 6-stage	5,707	940
	Air; Andersen 6-stage	3,622	1,279
Felling	Air; RCS-Plus,† 12–15 m above ground	906	294
	Air; RCS-Plus, 12–15 m above ground	881	213
Cutting limbs	Air; RCS-Plus, 6 m above ground	750	1,719
	Air; RCS-Plus, 6 m above ground	–	2,968
Cutting tree trunk	Air; RCS-Plus, 0–3 m above ground	21,250	225
Wood chipping	Air; RCS-Plus	53,125	21,250

*Limit of detection of method: 6 CFU/m^3^. †RCS, Reuter centrifugal sampler; limit of detection of method: 5 CFU/m^3^.

A log and a sample of wood chips from 1 of the removed trees were retained in the laboratory for 2 years, stored in sealed plastic bags at room temperature. Air samples taken close to the log after 1 and 2 years detected 25 CFU/m^3^ and <5 CFU/m^3^ *C. gattii*, respectively. Similarly, air samples taken at the opening of the wood-chips storage bag detected 2,256 and 1,494 CFU/m^3^ after 1 and 2 years, respectively, indicating long-term aerosolization of propagules from these tree byproducts. *C. gattii* was also isolated from 2 samples of wood chips (2,143 and 145 CFU/g) collected from within a wood chipping machine during a municipal cleanup of garden waste. Wood debris sampled directly from the chipper blade yielded 18 CFU/g of *C. gattii*, and an air sample taken at the outlet of the chipper yielded 19 CFU/m^3^ of *C. gattii*. Woodchips obtained from this service are used primarily to cover trails in the local parks (P. Crawshaw, 2003, pers. comm.). These data indicate that forestry activities and the distribution of tree byproducts may facilitate the mobility of *C. gattii* through both aerosolization and mechanical dispersal.

Residing within 10 km of sites of commercial soil disturbance or vegetation clearing has been reported as the most significant risk factor for *C. gattii* infection in domestic cats and dogs in BC (*5*). While we have not yet specifically tested the effect of soil disturbance on the aerosolization of *C. gattii*, we have observed that the highest concentration of *C. gattii* occurs within the top 15 cm of soil (Kidd et al., unpub. data) and could potentially be aerosolized through both large- and small-scale soil disturbances such as deforestation, landscaping and gardening, vehicles traveling on dirt roads, or rain splash (*17*).

### *C. gattii* Dispersal as a Model for Emerging Infectious Diseases

The emergence of *C. gattii* infection among humans and animals with no travel history to endemic areas raised the possibility of dispersal within the Pacific Northwest (*6*) and the observed colonization of *C. gattii* on wood products such as wood chips and mulch, in bodies of fresh water and seawater, in air, and in soil, suggests there could be several mechanisms for this dispersals (Kidd et al., unpub. data). The unique opportunity for investigation of *C. gattii* as an emerging pathogen in BC has facilitated insight into the ecology and distribution of this pathogen. We believe the dispersal mechanisms of *C. gattii* could be applied as a model for other organisms.

The mechanisms of *C. gattii* dispersal discussed here are similar to those described in a number of reports for other mammalian pathogens. For example, *Coccidioides immitis* is similar to *C. gattii* in that it primarily colonizes soil, and disease is acquired by inhaling aerosolized spores. Coccidioidomycosis outbreaks have occurred as a result of soil disturbances as well as windborne dispersal. Such outbreaks were documented after a California earthquake (*18*), after separate point-source exposures among archaeology students in northern California and Utah (*19*,*20*), and after a windstorm in Kern County, California, that led to many coccidioidomycosis cases in non–disease-endemic areas of the San Joaquin Valley in California (*21*).

*Blastomyces dermatitidis* is the cause of blastomycosis outbreaks in humans and animals in Wisconsin (*22*,*23*), and dispersal occurs by way of rivers (*24*–*26*). This dispersal is similar to the isolation of *C. gattii* from bodies of water in BC. *B. dermatitidis* infection has also been reported in 2 captive California sea lions in the adjacent US states of Wisconsin and Illinois (*27*), although no related environmental sampling was discussed in these cases.

We have compiled evidence that suggests human-mediated dispersal of *C. gattii* may be important, including the detection of multiple *C. gattii* strains in the wheel wells of vehicles and on footwear. Footwear and farm vehicles were found to be involved in mechanical transmission of porcine reproductive and respiratory syndrome virus in Minnesota (*28*). Similarly, contaminated footwear and vehicles have been implicated in the spread of foot and mouth disease, resulting in the establishment of strict disinfection protocols in certain parts of the world (*29*,*30*). In addition, dispersal of the oomycete plant pathogen *Phytophthora ramorum*, causing sudden oak death in North America and Europe, is mediated by human activity as well as natural factors (*31*–*33*).

A specific route of *C. gattii* introduction to the Pacific Northwest has not been established, although 1 hypothesis implicates importation of contaminated trees (*9*,*10*). The data presented here indicate that *C.* *gattii* could also have been introduced by mechanical vectors such as vehicles or footwear, or by wooden pallets or crates (*34*) that are not routinely inspected for microbial contamination upon entry into Canada. Bird and animal migration may be involved in *C. gattii* dispersal through passive transport as well. Certainly, a large number of migratory birds pass through the disease-endemic area on Vancouver Island.

Given numerous possibilities for dispersal of *C. gattii*, until more study is done on conditions favoring or inhibiting de novo colonization, attempts to restrict further dispersal of the organism would be impractical and unlikely to succeed. However, public health, medical, and veterinary personnel, as well as the public in the Pacific Northwest and elsewhere, must be made aware of *C. gattii* and symptoms of infection so the disease can be identified correctly and managed effectively.
